# Transcriptome of the Krushinsky-Molodkina Audiogenic Rat Strain and Identification of Possible Audiogenic Epilepsy-Associated Genes

**DOI:** 10.3389/fnmol.2021.738930

**Published:** 2021-11-04

**Authors:** Lyubov N. Chuvakova, Sergei Yu Funikov, Alexander P. Rezvykh, Artem I. Davletshin, Michael B. Evgen’ev, Svetlana A. Litvinova, Irina B. Fedotova, Inga I. Poletaeva, David G. Garbuz

**Affiliations:** ^1^Engelhardt Institute of Molecular Biology, Russian Academy of Sciences, Moscow, Russia; ^2^Moscow Institute of Physics and Technology, Dolgoprudny, Russia; ^3^FSBI Zakusov Institute of Pharmacology, Moscow, Russia; ^4^M.V. Lomonosov Moscow State University, Moscow, Russia

**Keywords:** audiogenic epilepsy, Wistar rats, KM rat strain, transcriptomic analysis (RNA-seq), *Ttr*, *Msh3*, MAPK signaling cascade

## Abstract

Audiogenic epilepsy (AE), inherent to several rodent strains is widely studied as a model of generalized convulsive epilepsy. The molecular mechanisms that determine the manifestation of AE are not well understood. In the present work, we compared transcriptomes from the *corpora quadrigemina* in the midbrain zone, which are crucial for AE development, to identify genes associated with the AE phenotype. Three rat strains without sound exposure were compared: Krushinsky-Molodkina (KM) strain (100% AE-prone); Wistar outbred rat strain (non-AE prone) and “0” strain (partially AE-prone), selected from F2 KM × Wistar hybrids for their lack of AE. The findings showed that the KM strain gene expression profile exhibited a number of characteristics that differed from those of the Wistar and “0” strain profiles. In particular, the KM rats showed increased expression of a number of genes involved in the positive regulation of the MAPK signaling cascade and genes involved in the positive regulation of apoptotic processes. Another characteristic of the KM strain which differed from that of the Wistar and “0” rats was a multi-fold increase in the expression level of the *Ttr* gene and a significant decrease in the expression of the *Msh3* gene. Decreased expression of a number of oxidative phosphorylation-related genes and a few other genes was also identified in the KM strain. Our data confirm the complex multigenic nature of AE inheritance in rodents. A comparison with data obtained from other independently selected AE-prone rodent strains suggests some common causes for the formation of the audiogenic phenotype.

## Introduction

Epilepsy is one of most common neurological diseases and up to 30% of all epilepsy cases are characterized by high pharmacoresistance. Many drugs used to treat epilepsy are characterized by severe side effects (Ornoy, 2009; Perucca and Gilliam, 2012; Chen et al., 2017). Accordingly, there is a need to develop new antiepileptic drugs, which requires the availability of valid laboratory models of epilepsy that permit investigation into the molecular mechanisms underlying epileptogenesis and evaluation of the effectiveness of new anticonvulsants. Several genetic models of epilepsy have been described, including rodent model strains. The development of convulsive seizures in response to intense sound (audiogenic epilepsy, AE) is induced in rodent models of epilepsy. In contrast to chemically induced or electroshock-provoked seizures, animals with the AE phenotype recover quickly after seizures and can be used as “self-controls” in long-term experiments with repeated provocation of seizures in the same animal (for reviews of different epilepsy models, see Bizière and Chambon, 1987; Kupferberg, 2001; Löscher, 2017). It should be noted that the so called refractory epilepsy is also present in the list of epilepsy forms in humans, most of these cases being also pharmacoresistant. The partial or generalized seizures in these cases occur in response to sensory stimulation (tactile, optical or acoustical, or even as a reaction to the certain mood of a patient). These cases are rather rare (about 6%) and not well analyzed, and there are only sparse indications concerning their genetic base (Xue and Ritaccio, 2006; Maguire, 2012; Taylor et al., 2013; Dinopoulos et al., 2014; Dewhurst et al., 2015; Sala-Padró et al., 2015; Atalar et al., 2020; Geenen et al., 2021). As for animal models the AE rodent strains used have certain benefits in comparison to chemical and electroshock models and they are widely used, as they permit to make chronical observations (Garcia-Cairasco et al., 2017). The genetic basis of AE development in rodents has attracted the attention of many groups (Ribak et al., 1988; Garcia-Cairasco, 2002; Poletaeva et al., 2017; Damasceno et al., 2018, 2020; Díaz-Casado et al., 2020). The data currently available generally support previously postulated hypotheses of polygenic inheritance of AE (Poletaeva et al., 2017), which is also characteristic of many forms of human epilepsy (Koeleman, 2018; Leu et al., 2020).

In the course of tonic audiogenic convulsion development (starting with the activation of acoustic nuclei in the medulla), the intense excitation process activates a specific brainstem neuronal network, in which inferior and superior colliculi (IC + SC) comprising the *corpora quadrigemina* play key roles. This activation was demonstrated in AE-prone rodent strains selected independently: GEPR (genetic epilepsy-prone rats, United States), WAR (Wistar audiogenic rats, Brazil), KM (Krushinsky-Molodkina, Russia), and in AE-prone GASH/Sal hamster strain (Faingold et al., 1992; Ribak et al., 1993; Faingold, 1999, 2002; Faingold and Casebeer, 1999; Garcia-Cairasco, 2002; Merrill et al., 2003; Doretto et al., 2009; Solius et al., 2016, 2019; Prieto-Martín et al., 2017; Ribak, 2017; Revishchin et al., 2018). It is now widely accepted that at the biochemical and physiological levels, AE proneness is associated with anomalies in brain GABAergic and glutamatergic systems, particularly in the *corpora quadrigemina* (Ribak et al., 1993; Korotkov et al., 2015; Solius et al., 2016; Chernigovskaya et al., 2019). The specific morphology of these brain stem structures was described in detail (Solius et al., 2016, 2019; Ribak, 2017; Revishchin et al., 2018), as well as the increased activity of the MAPK/ERK pathway in the striatum and hippocampus of KM rats (Dorofeeva et al., 2017; Chernigovskaya et al., 2019). Notably, in KM and GEPR rat strains, dopamine, norepinephrine, and serotonin brain levels significantly differed from those of control non-AE-prone animals (Poletaeva et al., 2017) and of histamine and adenosine system rats as well (Feng and Faingold, 2000; Vinogradova et al., 2007; Midzyanovskaya et al., 2016).

In this work, we investigated the profile of gene expression in the *corpora quadrigemina* consisting of two structures, inferior and superior colliculi (IC and SC) in three rat strains not exposed to sound (i.e., the “background” profile) to identify genetic traits associated with a hereditary predisposition to AE. It is known that an audiogenic seizure begins after an intense acoustic “startle reaction” with a phase of “wild running”, which is accompanied by clonic-tonic convulsions (Poletaeva et al., 2017). It was described previously (using different AE models) that the initial “ignition” of the seizure generating process is localized in IC, while the further generation of the wild run stage depends on the involvement of SC neuronal network. Both of these structures carrying several genetic anomalies, found in the best described AE rat models (Ribak et al., 1993; Faingold, 1999, 2002; Faingold and Randall, 1999; Doretto et al., 2009; N’Gouemo et al., 2009, 2010; Solius et al., 2016; Coleman et al., 2017; García et al., 2017; Ribak, 2017; Chernigovskaya et al., 2018; Revishchin et al., 2018). Nevertheless, the results of these and other works show that, although the beginning of the impulse in SC precedes that in IC, both of these brain stem structures are activated during the initial behavioral reactions in response to the sound stimulus. For example, in experimental work with damage of the deep layers of the SC (DLSC) in DBA/2 mice, there was a weakening of audiogenic convulsive seizures, but not a cessation of wild running (Willot and Lu, 1980; Tsutsui et al., 1992; Ribak et al., 1994). SC damage, including deep layers, in GEPR-3 and GEPR-9 rats did not stop the wild running phase, but weakened the severity of clonic-tonic convulsive reactions (Merrill et al., 2003). This work showed that the initial behavioral episodes (wild running or myoclonic tremors) are the only behavioral components of seizure in GEPR that persist after massive SC lesions. The results demonstrate that, although the beginning of the impulse in SC precedes that in IC, both of these brain stem structures are activated during the initial behavioral reactions in response to the sound stimulus. To date, it has been established that the progression from wild running to the occurrence of convulsive reactions is slowed down by injuries at both the IC and SC levels. The interruption of the connection between IC and SC reduced the severity of sound-induced seizures in sound-sensitive Wistar and GEPR-9 rats (Tsutsui et al., 1992; Ribak et al., 1994; Merrill et al., 2003). In addition, the involvement of IC in the initiation of the wild running phase is also demonstrated by the fact that unilateral electrical stimulation of the rostral part of the external IC core (ICx) in a normal (non-AE-prone) rat causes a wild running behavior component (Ribak et al., 1994; Chakravarty and Faingold, 1996). These data show that pathways from the IC to the SC play a role in the spread of the audiogenic seizures, although the details of “intra-quadrigeminal” interactions for norm and AE pathology are not well known. Thus we concluded that the separation of IC and SC structures for our initial connectome analysis could damage the internal connections between these nuclei (as the axons from IC could contain the RNA molecules, which play certain role in case of sound exposure, i.e., are important for the subsequent excitation of SC). Thus the separation of these two paired structures could affect the results, making them less convincing. This was the ground for our choice to monitor the molecular events in these two structures cumulatively.

We used the inbred Krushinsky-Molodkina (KM) strain, which was selected for AE proneness in the late 1940s from the Wistar outbred population. The KM strain has a history of more than 40 generations of brother-sister inbreeding. Almost 100% of the individuals in this strain develop a characteristic epileptiform seizure 3–4 s after the onset of sound exposure (120 dB intensity), which is first manifested with a “wild run” phase, quickly followed by clonic and tonic seizures (Poletaeva et al., 2017). For controls, we used non-AE-prone rats of an outbred Wistar strain and “0” strain rats, based on an initial F2 (KM × Wistar) hybrid population, first selected in 1998 for their non-AE-prone phenotype. This selection aimed to create a non-AE-prone strain with a genetic background closer to that of KM rats than that of current Wistar rats (Fedotova et al., 2012; Poletaeva et al., 2014, 2017). In the “0” strain population, the proportion of rats without AE ranges from 30 to 60%. According to lab protocol these animals had been exposed to sound three times at the age of 3 months and respective proportions of AE-non-prone animals were found, the exact proportions varies in generations. Thus, by comparing the transcriptomes of these three strains, we were able to identify the peculiarities of gene expression associated with both the development of the AE phenotype (as a result of the selection of KM rats from the Wistar rat population) and the respective changes (i.e., the type of “compensation”) in the initial AE phenotype exhibited in the KM rats as a result of “0” strain selection.

Previously, limited data were obtained for transcriptomes of the following other audiogenic rodent strains: WAR, selected from Wistar rats in Brazil (independent of the KM rat population), and AE-prone GASH/Sal, a hamster strain (Damasceno et al., 2018, 2020; Díaz-Casado et al., 2020; Díaz-Rodríguez et al., 2020). Thus, the gene expression profile of the *corpora quadrigemina* in KM rats may be compared with that of other AE-prone animals.

In the present study, we were able to identify characteristic features in the *corpora quadrigemina* transcriptome in KM rats and differentiate them from those in the Wistar strain and, to a lesser extent, from those associated with the transcriptome in the “0” strain. The results obtained revealed a number of gene expression profile features that may be associated with AE. These changes included those at the transcription level of the *Ttr* gene, which is involved in the regulation of the expression of GABAergic receptors (Zhou et al., 2019), and in the *Msh3* gene, which encodes a component of the DNA repair system (Park et al., 2013). Moreover, KM rats showed increased expression of several genes, the products of which are involved in the positive regulation of the MAPK signaling cascade, as well as in phosphorylation of ERK1/2, which may be involved in the development of the AE-prone phenotype (Nateri et al., 2007; Glazova et al., 2015; Dorofeeva et al., 2017). Several genetic traits that are plausibly connected to the development of AE are universal, as they were discovered in two distant rodent species (rats and hamsters), while others are inherent for definitive strains that were bred independently (i.e., KM and WAR rats) (Damasceno et al., 2020). In general, the data obtained reveal the complex polygenic nature of AE-prone rodents.

## Materials and Methods

### Animals

The brain tissues of 4-month-old male rats grouped into three genotypes (Wistar, KM, and “0”) were used. The animals were maintained in standard plastic cages (T4) with *ad libitum* food (Lab Feed) and water and a natural light-dark schedule. The manifestation of AE seizures was tested in a transparent plastic sound-attenuating chamber (“Open Science”) using a standard classroom bell (sound intensity of 120 dB). For the KM rats, the maximum intensity of the tonic convulsions in response to sound (score of “4,” according laboratory classification) was recorded (Poletaeva et al., 2017).

Herein, we used Wistar rats and rats of selected “0” strain, as they demonstrate no or moderate seizures in response to sound. According to lab protocol at the age of 3 months KM rats were sound exposed to verify their seizure reaction (tested once only). All “0” strain animals were exposed three times at the same age as KMs. This “triple” test was needed in order to verify the real lack of their reaction to sound. This strain is still under selection and non-proneness of these animals is not 100%. In the previous selection generations only 30–60% of “0” strain animals had no AE seizures. The lab protocol for genetic selection is triple AE testing for “0” rats in order to be sure that there were no minor AE signs (Fedotova et al., 2012). Wistar rats were tested for AE 4 days before sacrificing, while KM and “0” rats were also exposed to sound at the same time in order to equalize the physiological status of all three groups. The 4 day interval was considered to be enough for negative reactions to wane, and there were no changes noted in animal behavior before sacrificing. The strain “0” rats are under selection for AE non-proneness F2 (KM × Wistar) hybrids being their initial population. In F2 and in further generations animals differed in their reaction to sound from the total lack of audiogenic seizures to clonic-tonic fit. In F41 selection generation the animals tested demonstrated only wild run AE stage.

In 1940–1950s KM rats (as well as later Wistar Audiogenic Rat strain from Brazil, Garcia-Cairasco et al., 2017) were selected from Wistar outbred population. About 15% of Wistar rats usually demonstrate the moderate AE proneness, wild run stage and (rarely) clonic convulsions. In our experiment Wistar rats were tested for sound sensitivity in the same way as KM rats. The few animals that showed the “wild run” phase were excluded from the further experiment. Thus, neither “0,” nor Wistar rats, used in this study, were AE-prone. At the same time Wistar and “0” rats had distinct acoustic startle-response, indicating that they were not deaf.

The inferior and superior colliculi (IC + SC) of *corpora quadrigemina* were isolated from animals 4 days after sound exposures, in order not to compromise gene expression profile with seizure-mediated transcriptional changes, as we consider that 2 day interval [acquired in Damasceno et al. (2018)] are not enough for elimination the plausible metabolic consequences of seizure induction and/or of sound exposure (in cases when no seizure occurs). Euthanasia was carried out by rapid guillotine decapitation (“Open Science”). After euthanasia, the brains were quickly extracted in the cold and the fraction of the brain stem which contained IC + SC was isolated. The isolated brain tissue samples were frozen and stored in liquid nitrogen.

The experimental procedures were performed in accordance with requirements of Declaration 2010/63/EC.

### RNA Extraction, Preparation of RNA-Seq Libraries and Sequencing

For RNA extraction, IC + SC sections were isolated from the brains of 4-month-old KM (*n* = 3), Wistar (*n* = 4), and 0 (*n* = 4) rats. Total RNA was extracted using Extract RNA reagent (Evrogen). The concentration of RNA was measured with a Qubit fluorometer (Invitrogen). The quality of the RNA was determined with an Agilent BioAnalyzer 2100 using an RNA 600 nano kit. The RNA integrity number (RIN) of all the RNA samples was not less than 8. Poly(A)-enriched libraries were prepared for RNA-seq using the NEBNext Ultra II Directional RNA Library Prep Kit for Illumina according to the manufacturer’s protocol. Seventy-five-bp single-end sequencing was conducted on an Illumina NextSeq 500 platform.

### RNA-Seq Analysis

As a result of deep sequencing, we obtained 15–22 million reads of RNA for each library. Preprocessing of these data was performed using a PPLine script that includes Trimmomatic for the adapter, length and quality trimming, STAR for mapping reads to the rat genome (release Rnor_6.0), and HTSeq-count for calculation of the read counts aligned on genes (Dobin et al., 2013; Bolger et al., 2014; Anders et al., 2015; Krasnov et al., 2015). A differential expression analysis was performed with the edgeR package using a quasi-likelihood negative binomial generalized log-linear model (QLF test) (Robinson et al., 2010). Gene Ontology and KEGG enrichment analyses were performed using the topGO (v.2.42.0) and clusterProfiler packages (Yu et al., 2012; Alexa et al., 2020). For GSEA analysis only differential expressed genes (*p* < 0.05) were used. The differential expression analysis, visualization, and GSEA were performed using RStudio program and ggplot2 package, as well as custom scripts written in Python (Wickham, 2009).^1^ A Venn diagram was generated using FunRich (Pathan et al., 2015).

Compensation coefficients of the differentially expressed genes in the “0” rat samples compared to those in the KM rats were calculated using the equation (mean (logCPM) KM – mean (logCPM) 0)/(mean (logCPM) KM – mean (logCPM) W). The density distribution of genes was estimated in an R environment using kernel density estimation (KDE).

The RNA-seq data reported in this study can be accessed using GEO accession number GSE173885.

### Immunoblotting

Samples from six animals of each strain were used for the immunoblotting series. Total protein extracts of the IC + SC tissue samples were prepared using the ReadyPrep protein extraction kit (Total Protein) (Bio-Rad) according to the manufacturer’s recommendations upon addition of phosphatase inhibitor cocktail (Sigma). Thereafter, the protein samples were homogenized in SDS-PAGE buffer (65 mM Tris–HCl, pH 6.8; 10% glycerol; 2% SDS; 1% DTT; and 0.01% bromophenol blue) and heated to 100°C for 5 min. Electrophoresis was performed in 10% PAAG, and the proteins were transferred to a Hybond ECL membrane (GE Healthcare) by the semidry technique with buffer containing 25 mM Tris, 0.2 M glycine, 20% methanol, and 0.01% SDS with a current power of 1.5–2 mA/cm^2^ for 1.5 h. After protein transfer, the membrane was blocked by 5% ECL blocking agent (GE Healthcare) in TBST (20 mM Tris–HCl, pH 7.6; 0.14 M NaCl; and 0.01% Tween 20) for 1 h. After blocking, the membrane was incubated for 12 h with primary anti-phospho-p44/42 MAPK (ERK1/2) (Thr202/Tyr204) polyclonal rabbit antibodies (9101, Cell Signaling) to detect phospho-ERK1/2 and anti-p44/42 MAPK (ERK1/2) polyclonal rabbit antibodies (9102, Cell Signaling) to detect total ERK1/2 at the dilutions recommended by the manufacturer. Next, the membranes were washed four times for 10 min each time with TBST and incubated for 1 h with horseradish peroxidase-conjugated secondary anti-rabbit antibodies (A0545, Sigma-Aldrich) at the dilution recommended by the manufacturer. To remove bound primary and secondary antibodies and reprobe the proteins, the membranes were washed with Restore PLUS Western blot stripping buffer (Thermo Fisher Scientific). For signal detection, the SuperSignal West Pico PLUS chemiluminescent substrate (Thermo Fisher Scientific) was used. The signal intensities were measured with a gel-documenting system, ChemiDoc MP, and the Quantity One 1-D analysis software program (Bio-Rad). The optical densities of the bands were analyzed in the quantized image with the ImageJ program. Phospho-ERK1/2 signals were normalized to the signals obtained for total ERK1/2.

### Quantitative PCR

Four-month-old KM (*n* = 9) and Wistar (*n* = 10), and “0” (*n* = 9) rats were used sampled for PCR analysis. Total RNA extraction from the *corpora quadrigemina* was performed using RNAzol RT (Molecular Research Center, United States). The concentration of isolated total RNA was measured with a NanoDrop spectrophotometer (Thermo Fisher Scientific, United States). cDNA was prepared from 1 μg of total RNA using random primers and MMLV reverse transcriptase (Evrogen, Russia). The analysis was performed on an ABI PRISM^®^ 7500 sequence detection system (Applied Biosystems, United States). Detection of amplification products was carried out using SYBR Green 1 in the presence of ROX reference dye (Evrogen, Russia) according to the manufacturer’s recommendations. Expression levels of genes of interest were normalized to the expression of the *Ywhaz* housekeeping gene (Schwarz et al., 2020) and calculated using the 2^–*dCt*^ equation. The sequences of the primers used are shown in Supplementary Table 1.

### Statistical Analysis

Non-parametric ANOVA (Kruskal-Wallis test with *post hoc* analysis) was used to compare qPCR results and Western blot signals, respectively. *p*-values < 0.05 were considered statistically significant.

## Results

### The Observed Inter-Strain Differences in Gene Expression Profiles Indicate the Multifactorial Nature of Audiogenic Seizures

**FIGURE 1:**
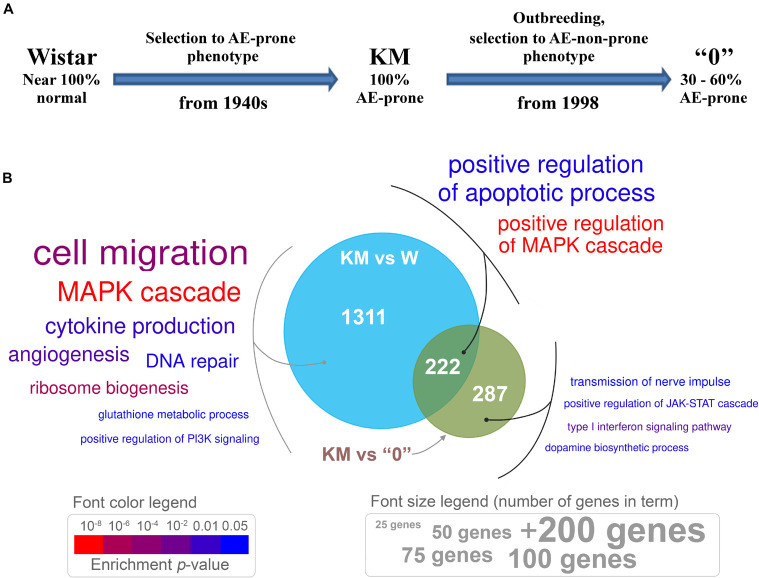
**(A)** The sequential procedure for deriving the Krushinsky-Molodkina (KM) and “0” strains from the Wistar strain. **(B)** Venn diagram depicting differentially expressed genes (DEGs) between KM samples and those from W and “0” rats, which exhibit distinct genotypes. Genes with differential expression, *p* < 0.05, and expression changes greater than 1.5-fold (either up- or downregulated) were used for enrichment analysis. Enriched Gene Ontology (GO) terms are presented in colored text. The font size is proportional to the number of genes in a term (see the “font size legend”). Font color indicates the enrichment test *p*-value (see the “font color legend”).

As mentioned above, aiming to reveal the molecular mechanisms of AE, the gene expression profiles of KM rat *corpora quadrigemina* were compared with those of Wistar strain (W) rats, which do not exhibit AE, and “0” strain rats selected from KM × Wistar hybrids for the absence of AE phenotype (Figure 1A).

Differential gene expression analysis revealed 1488 genes [DEGs, *p* < 0.05, quasi-likelihood *F*-test (QLF test)] in the transcriptome of the KM samples compared to the transcriptomes of age-matched W rats. The transcriptome of “0” rat samples showed fewer differences from that of the KM rat samples (494 DEGs, *p* < 0.05, QLF test; Supplementary Table 2). This result (fewer differences in the KM rats vs the “0” rats, compared to those found in KM vs W) is in accordance with the possible role of two factors. The first factor is related to the relatively recent appearance of a non-AE-prone phenotype in the course of “0” rat strain selection (fewer than 40 generations), and the second factor is related to the influence of the KM rat genotype on that of the “0” rat strain, which revealed the “hidden” AE proneness of the “0” rats (i.e., proneness not evident in the normal state of these animals). At the initial stages of “0” strain rat selection, several backcrosses to the KM parental rat strain had been performed (Fedotova et al., 2012) in order to obtain animals with a genetic background more similar to that of KM rats as currently existing Wistar rats differ from KM rats, due to the history of long separate breeding. This “hidden” AE proneness was revealed first, as not 100% of the “0” rats were non-AE-prone, and then, as expected, the AE seizures in the “0” rats developed after induction with a subthreshold dose of penthylenetetrazole (Fedotova et al., 2016; Poletaeva et al., 2017). In accordance with this finding, we used only the rats in the “0” rat strain group that did not respond to sound (see “Materials and Methods” section).

To annotate functional gene expression differences in the three rat genotypes, a comparative analysis of genes with altered expression levels was performed with KM, W, and “0” strain rats (the first strain being highly AE-prone, 100% of animals from two other groups were non-seizure prone). The results are discussed in terms of the possible involvement of these genes in neurological processes (Gene Ontology) and molecular networks (KEGG pathway enrichment).

The Venn diagram (Figure 1B) demonstrates the number of DEGs in the KM rat IC + SC brain tissue samples compared with those in the W and “0” rat tissue samples. Genes with expression levels that differed between the KM and W rats are involved in various processes, including cell migration, cytokine production, ribosome biogenesis, glutathione metabolism, regulation of PI3K signaling, DNA repair and the MAPK cascade (Figure 1). These results are not surprising, as KM and W rats were bred separately for more than two hundred generations, starting at the end of the 1940s. Selection of the “0” rat strain from KM × W hybrid rats resulted in differences that affected the expression of genes participating in dopamine biosynthesis, nerve impulse transmission, type I interferon signaling and regulation of the JAK-STAT cascade (Figure 1). Interestingly, genes common to the two groups that were compared (KM vs W rats and KM vs “0” rats) are involved in the regulation of the MAPK cascade and apoptotic process (Figure 1).

**FIGURE 2:**
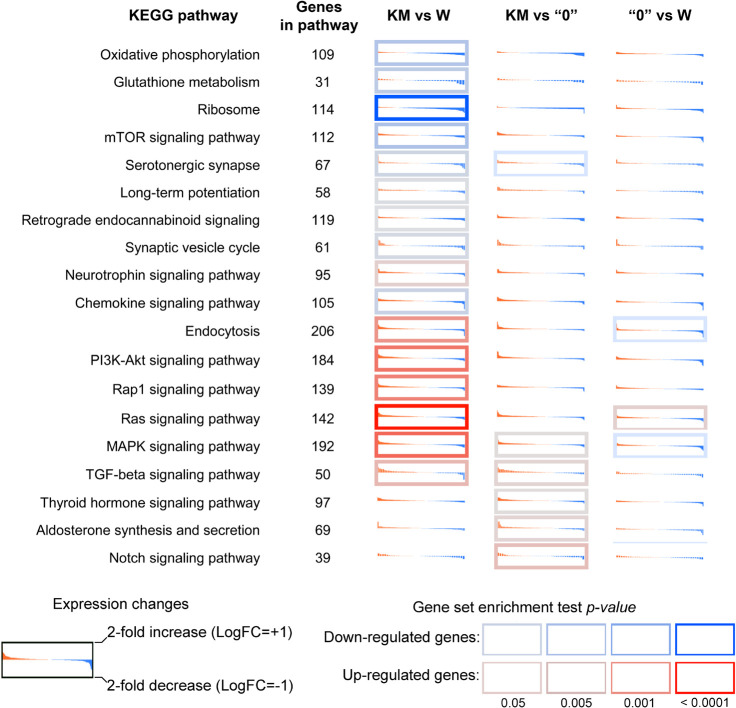
Differential expression profiles of genes participating in the most affected KEGG pathways in the KM, W, and “0” rats. For each KEGG term, genes were identified (irrespective of *p*-value) and sorted by decreasing log2-fold change. Red-to-blue sub-plots illustrate expression level changes (log-scale, with values decreasing across the plot). The log2 expression level fold change (LogFC) range extends from −1 (i.e., 2-fold downregulation; blue) to +1 (i.e., 2-fold overexpression; red). The cell border indicates the gene set enrichment (GSEA) *p*-value for a pathway. A red border indicates that a KEGG pathway is enriched with upregulated genes; a blue border indicates that a KEGG pathway is enriched with downregulated genes. The *p*-value was calculated for up- and downregulated genes using Fisher’s exact test.

Analysis of enrichment of DEGs in KEGG pathways showed that differences in the gene expression profile included the downregulation of ribosome biogenesis, glutathione metabolism, the mTOR pathway, oxidative phosphorylation, glutathione metabolism, serotonergic synapse, and the synaptic vesicle cycle in the KM rats compared with the W rats (Figure 2). The upregulated pathways in the KM rats included the PI3K-Akt, Rap1, Ras, and MAPK signaling pathways (Figure 2). The comparison of KM and “0” rat samples revealed the upregulation of aldosterone synthesis, thyroid hormone and Notch signaling pathways in the KM rats (Figure 2). Finally, the transforming growth factor β (TGFβ) signaling pathway was upregulated in the KM rats compared with both the W and “0” rats (Figure 2).

These data indicate that breeding of the KM strain induced significant gene expression profile changes in comparison with that of the Wistar rats, and the latter can be regarded comprising the initial population. The selection for increased sound reactivity among successively bred KM strain rats, including seizure provocation, resulted in “shifts” toward a presumably pathological state, which affected different processes. This selection affected synaptic plasticity, cytokine production, the DNA repair system and key neurochemical pathways, including signal transmission *via* different kinases (MAPK and PI3K). It is reasonable to suggest that the “0” strain non-AE-prone phenotype is probably the result of changes in MAP kinase activity, dopamine synthesis and type I interferon signaling, which may be crucial for decreasing AE proneness.

### The Mitigation of ERK1/2 Activity and Changes in the Expression of Genes Involved in the MAP Kinase Pathway Are Characteristics of the Non-AE-prone Phenotype

Extracellular signal-regulated kinases 1 and 2 (ERK1 and ERK2) are essential components of the classical pathway of MAP kinase, and they are believed to be among the factors that mediate epileptiform seizures, at least in *Fmr1*-KO mice and KM rats (Nateri et al., 2007; Curia et al., 2013; Glazova et al., 2015; Dorofeeva et al., 2017). Different regional distributions of ERK1/2 in KM and W rats and higher levels of phosphorylated ERK1/2 (i.e., the active form) in KM rats were recently demonstrated (Dorofeeva et al., 2017; Chernigovskaya et al., 2019). In accordance with these data, the differential gene expression pattern of MAPK pathway participants in this study was shared by comparisons of two pairs (KM vs W rats and KM vs “0” rats) (Figure 1). This observation indicates that expression changes in components of the MAP kinase cascade distinguish KM rats from both W and “0” rats (Figure 1).

**FIGURE 3:**
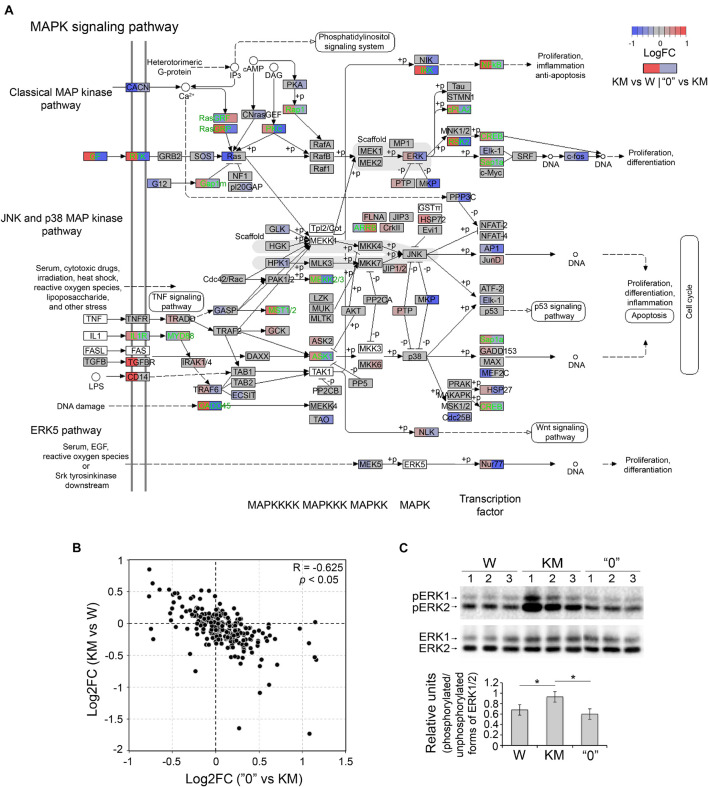
The expression of MAPK pathway and ERK1/2 genes was alleviated in “0” rats. **(A)** Differential expression of genes participating in the MAPK pathway (rno04010). Gene expression changes are shown in color (log2-scale; LogFC; red – upregulation, blue – downregulation, gray – no change, white background – the absence of expression). Each gene block is divided into two sections (the left block shows differential expression between KM and W rats; right – between “0” and KM samples). Genes that demonstrate opposite changes between two pairs of comparisons (“compensated” genes) are shown in green font. **(B)** Scatter plot depicting the pairwise comparison of log2-fold changes between the KM vs W and “0” vs KM groups. R – Spearman’s correlation coefficient. **(C)** Western blot analysis of phosphorylated and unphosphorylated forms of ERK1/2 (*top*) and the calculation of the optical density of the signals (*bottom*). For semiquantitative estimation, the optical density of phosphorylated ERK1/2 was subjected to two levels of normalization: first between samples and second on the unphosphorylated form of ERK1/2. *p < 0.05, Kruskal-Wallis test with *post hoc* analysis.

The detailed analysis of genes involved in the KM-specific MAPK cascade revealed the upregulation (in comparison with the expression in W rats) of classical MAP kinase pathway components, including the following genes: receptor tyrosine kinase (*RTK*) and RTK ligand, epidermal growth factor (*EGF*), nucleotide exchange factor RasGRP, and cyclic AMP-dependent transcription factor ATF-4, also known as CREB (Figure 3A). The KM rat gene expression profile was also characterized and compared to that of the W rats, and higher expression levels of interleukin 1 receptor (*IL1R*) and myeloid differentiation primary response-88 (*MYD88*) genes, as well as growth arrest and DNA damage (*GADD45*) genes and downstream agents of the p38 MAP kinase pathway were found. The latter observation suggests that neural cells of KM rats are highly susceptible to stress signals due to cytokine stimulation and DNA damage (Figure 3A; Mikhailov et al., 2005). Importantly, the expression changes of the aforementioned genes in “0”, when compared to KM samples, demonstrated the opposite expression pattern (Spearman’s correlation = −0.625, *p* < 0.05), which suggests that MAPK signaling features characteristic of KM rats are “compensated” in some manner in “0” rats (Figures 3A,B). This observation was supported by Western blot analysis, through which we determined the quantity of the phosphorylated (active) forms of ERK1/2 in W, KM and “0” rat samples. The results showed the increased levels of phosphorylated ERK1 and ERK2 observed in the KM rats samples compared with those in the W rat samples (increased by 40%, *p* < 0.05, Kruskal-Wallis test with *post hoc* analysis), and these levels were lower in “0” rats, resembling the levels intrinsic to W rats (Figure 3C).

Considering these results, we hypothesized that this “compensation” of the expression levels of genes implicated in the MAPK cascade is one of the factors contributing to the acquisition of the non-AE-prone phenotype in “0” rats.

### Non-AE-proneness Is Accompanied by the “Reversion” of the Expression Pattern of More Than One-Half of the Genes, Resembling That of the Progenitor State

**FIGURE 4:**
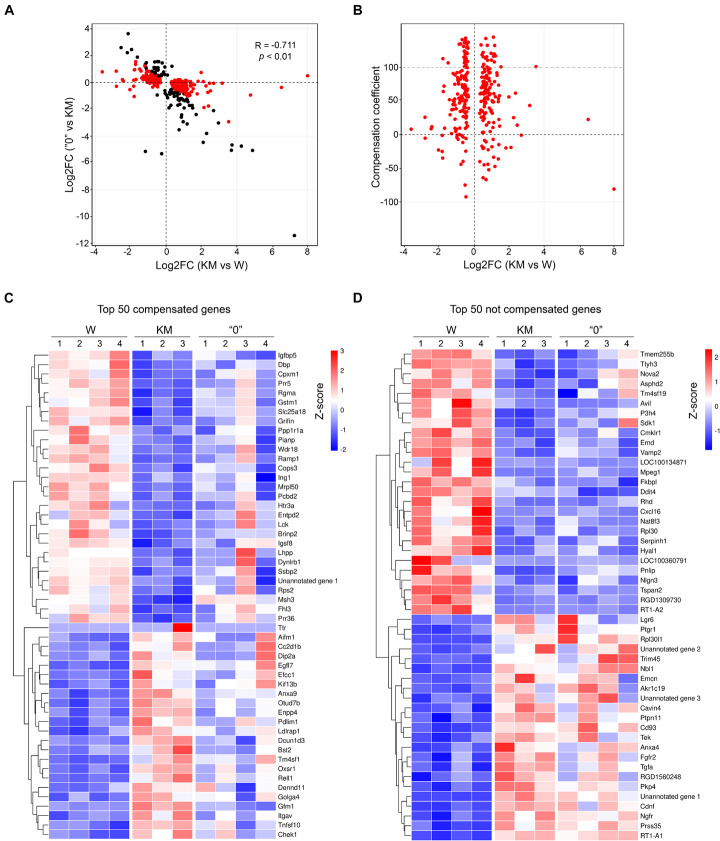
The expression levels of most genes in the “0” rat samples tended to be reverted to the levels in the W rats. **(A)** The log2-fold change of gene expression that shows the significant difference in the expression levels between the KM and W rat transcriptomes (X-axis) and between the “0” and KM rat transcriptomes (Y-axis). Positive values correspond to activation in the KM or “0” rats. One point represents one gene, and only genes exhibiting significant changes (*p* < 0.01, QLF test) are shown. Genes with differences in expression between the KM and W transcriptomes are shown in red, and those with differences only between the “0” and KM transcriptomes are shown in black. **(B)** Dependence of the compensation coefficient (Y-axis) on the log2-fold change in the KM and W rat comparison (X-axis). The compensation coefficient was calculated as a percentage of mean (logCPM) KM – mean (logCPM) 0)/(mean (logCPM) KM – mean (logCPM) W. **(C)** Heat map of the top 50 compensated genes (>50 of the compensation coefficient value) displayed in line from the highest *p*-value at the top to the lowest *p*-value at the bottom. **(D)** Heat map of the top 50 genes exhibiting the least or no compensatory expression (<50 of compensation coefficient value) displayed in line from the highest *p*-value at the top to the lowest *p*-value at the bottom. The expression values of the genes presented in panels **(C,D)** are Z-transformed.

The emergence of differences in genetic patterns (and thus in gene expression profiles) of KM and “0” rats was analyzed according to the strain selection history, which followed two stages. In the first selection stage, the AE-prone phenotype was acquired by KM rats. This development proceeded through several intermediate stages, which reflect the gradual increase in AE proneness (both penetrance and expressivity) during dozens of generational selections and resulted in nearly 100% AE proneness in the current KM strain. The second stage involved the selection of non-AE-prone animals from KM × W rat hybrids. Given the multifactorial nature of the AE-prone phenotype, it is logical to assume that selection for the non-AE-prone phenotype among rats in the W × KM hybrid population resulted in the selection of gene alleles with an expression pattern characteristic of non-AE-prone Wistar rats. To test this assumption, we compared the expression levels of genes with the greatest significant differences between the KM and W rats and between the KM and “0” rats (*p* < 0.01). This comparative analysis showed negative correlations of log2-fold changes (Spearman’s correlation = −0.711, *p* < 0.01), with over 70% of the DEGs exhibiting an opposite direction of expression, indicating that the pathophysiological genetic peculiarities in the KM rats had been lost in the course of strain “0” selection (Figure 4A).

To quantify this putative “compensation” effect in the gene expression profile in the “0” strain rats, we calculated the compensation coefficient as described in the “Materials and Methods” section. According to the formula used for the calculation, in cases when the compensation coefficient was close to 100% (i.e., in the “0” rat samples) almost complete “compensation” was observed for genes that showed different expression patterns in the KM in comparison with the W rats. Higher values of the compensation coefficient corresponded to overcompensation, and negative values corresponded to cases for which no compensation or enhancement of the KM phenotype was evident. Applying this approach, we showed that the expression levels of a large fraction of DEGs observed in the comparison of the KM and W rat samples were compensated (the compensation coefficient was between 1 and 100 for as many as 70% of the DEGs, with the most-significant changes indicated by *p* < 0.01; Figure 4B and Supplementary Figure 1). These data also indicate that the gene expression profile in the “0” rat strain was substantially “reversed” (i.e., “returned”) to that representing original expression levels, which were observed in the *corpora quadrigemina* of the W rats. Importantly, these “compensation data” were revealed for both upregulated and downregulated expression patterns, which distinguished the KM rat samples from the W rat samples) (Figure 4C). However, at least 15% of the DEGs that were predominantly downregulated in the KM rats (compared with the W rats) were not compensated in the “0” rats (Figure 4D). This finding may signify that the “0” rat strain carries part of the KM rat genetic background (the “0” rat strain can be induced to AE proneness with subthreshold penthylenetetrazole injection, as explained above).

**FIGURE 5:**
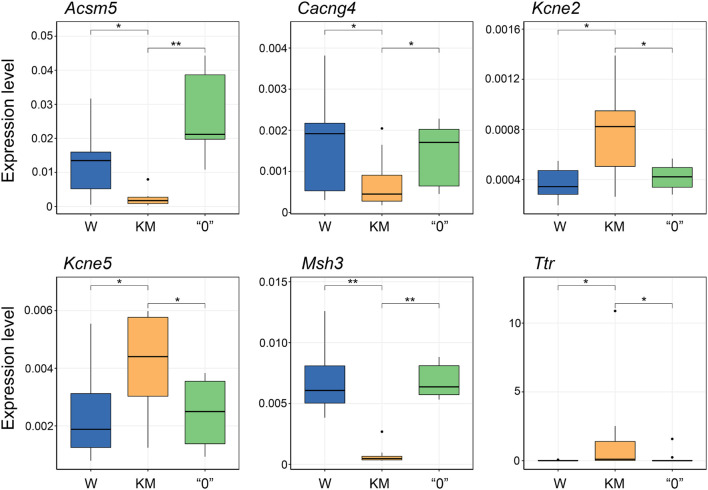
qPCR validation of gene expression changes initially observed through RNA-seq analysis. Expression levels determined by the 2^–*dCt*^ equation, including normalization of the genes of interest levels to a housekeeping gene (*Ywhaz*) for each group of samples. Expression levels were estimated for no fewer than 6 rats. *p < 0.05, **p < 0.01, Kruskal-Wallis test with *post hoc* analysis.

To validate the gene expression patterns revealed by RNA-seq, we performed qPCR analysis for six selected genes exhibiting opposite expression differences between the KM and “0” rat samples in comparison with the pattern of differences in the KM and W rat sample pairing. These genes evaluated were acyl-CoA synthetase medium-chain family member 5 (*Acsm5*); calcium voltage-gated channel auxiliary subunit gamma 4 (*Cacng4*); potassium voltage-gated channel subfamily E regulatory subunits 2 and 5 (*Kcne2* and *Kcne5*); mutS homolog 3 (*Msh3*), a component of the DNA mismatch repair system; and the transthyretin (*Ttr*) gene, which encodes the protein that transports thyroxin and the retinol-binding protein complex (Fishel, 1998; Park et al., 2013; Sharma et al., 2019). This analysis confirmed the differences in KM rat gene expression compared with W rat gene expression, as well as the trend in the “0” rat strain of gene expression level “reversion” to the levels observed in the W rats (*p* < 0.05, Kruskal-Wallis test with *post hoc* analysis, Figure 5).

In addition, in the comparison between KM and W rats, a decrease in the expression level of genes encoding components of the 1st, 3rd, and 4th complexes of the respiratory chain and of the F-ATPase genes was observed. In the “0” rats, compensation for the expression of the genes encoding 1st and 4th complexes and ATP synthase was detected, but not for the 3rd complex (Supplementary Figure 2).

Taken together, these results indicated that the selection of the “0” rat strain resulted in the large “reversion” in the gene expression profile toward that intrinsic to the progenitor W rats.

Thus, the genes that tended to “compensate” for the expression level in the “0” rat strain (see above) can presumably be considered genes involved in the development of the AE phenotype in the KM strain. Among these genes, we focused primarily on the genes involved in human epilepsy (i.e., their mutations and/or changes in the expression level of these genes in humans increase the risk of epilepsy). The DEGs in other rodent model strains with an AE phenotype were also of interest in this analysis, in particular, insulin-like growth factor-binding protein 5 (IGFBP5), which has been shown to associate with four and a half LIM domains protein 2 (FHL2), which tunes neuronal functioning (including seizure proneness) via androgen production and respective receptors (Reddy, 2004; Jamali et al., 2006). These genes also included glutathione S-transferases (GSTs), which conjugate electrophilic compounds and thus may play neuroprotective roles by removing exogenous and endogenous oxidants. Carriers of GSTM1-null genotypes in European and Asian populations have been found to show significant risk of developing epilepsy, emphasizing the involvement of the antioxidant system in seizure susceptibility (Ercegovac et al., 2015; Prabha et al., 2016). In our study, we found downregulation of *Igfbp5* and *Gstm1* gene expression in the KM rats. The abnormal pattern of the brain antioxidant system in KM rat strain has been previously demonstrated (Medvedev et al., 1992). The changes detected in the expression of the *Msh3* and *Ttr* genes in our study are also of interest because mutations or changes in the expression levels of these genes had been previously detected in other AE-prone rodent strains (WAR rats and GASH/Sal hamsters) (Damasceno et al., 2020; Díaz-Rodríguez et al., 2020).

## Discussion

Analysis of the transcriptome in audiogenic rat strain (KM) and its comparison with the transcriptome of non-AE-prone rats in the original Wistar population and with “0” rats subjected to “reverse” selection allowed us to identify candidate genes for which expression changes are presumably the cause of AE phenotype acquisition. This approach met with certain difficulty, as some of the differences detected (1488 DEGs in the KM vs W rats and 494 DEGs in the KM vs “0” rats) may have been the result of changes in the genetic background of the strains, which was noted in both the KM and Wistar rats due to random changes occurring during the separate breeding of KM and W rat strains over many generations. We assumed that seizure proneness (including AE proneness) emerged during the early stages of brain development. If this supposition is accurate, then an analysis of the adult animal brain transcriptome would not lead to the detection of key changes in gene expression that are critical for AE phenotype development from the early ontogeny to the age of 3 months. A previous analysis of AE development in KM rat ontogenesis showed that AE seizures are reliably (at full intensity) expressed at the age of 3 months (Fedotova and Semiokhina, 2002; Poletaeva et al., 2017). Similar dynamics were described for GEPR AE expression (Thompson et al., 1991). In accordance with these observations, we used 4-month-old rats in experiments (see “Materials and Methods” section). Notably, a direct comparison of gene expression patterns in rats and humans is not appropriate because of the immense differences in brain structures. Nevertheless, the comparison of our transcriptomic data for the set of rat strains (W, KM, and “0” strains) with data obtained for other rodent AE-prone animals, such as WAR rats and GASH/Sal hamsters (Damasceno et al., 2020; Díaz-Rodríguez et al., 2020), allowed us to identify patterns that are apparently causally related to the development of AE. The observed partial reversion of the gene expression profile in the “0” rats (partially non-AE-prone but derived from KM rats) toward the expression profile of the W rats and the ability to compare our results with other AE rodent models allowed us to reduce the number of genes of interest.

Considering our data, we concluded that the most characteristic feature of the KM rat IC + SC transcriptome is ∼10-fold decrease in the expression of the *Msh3* gene (MutS homolog) in comparison with that in the W and “0” rats (Figure 5). Along these lines, it was previously shown that the GASH/Sal AE-prone hamster strain carries a mutation in the *Msh3* gene potentially capable of disrupting Msh2/Msh3 protein heterodimerization (Díaz-Casado et al., 2020). These proteins were shown to form the MutSß complex, which participates in DNA repair by recognizing insertion-deletion loops of 1–15 nucleotides and DNA with 3′ single-stranded overhangs (Gupta et al., 2011); the authors suggested that the increase in *Msh3* expression level observed in GASH/Sal hamsters is probably due to changes in compensatory gene expression (Díaz-Casado et al., 2020). Disruption of the MutSß complex in mice with Msh2 deficiency was reported to induce a decrease in the expression levels of cytochrome *c* oxidase subunit 2 (CoxII), the ATP synthase β-subunit, and superoxide dismutase genes (in comparison with those in WT animals) (Francisconi et al., 2006). This genotypic change increased animals’ susceptibility to kainic acid (KA)-induced seizures. A single KA injection increased seizure proneness and hippocampal neuronal death, while systemic KA injections increased the mortality rate together with an increase in tonic-clonic seizure severity (Francisconi et al., 2006). The data previously reported also showed that disruption to the DNA repair system can cause mitochondriopathy in the brain, which presumably leads to predisposition for convulsions. As mentioned above, the gene expression profile of the KM rats compared to the W rats in our study demonstrated a decrease in *Acsm5* gene expression (Figure 5) and in a number of genes encoding components of the 1st (NADH:ubiquinone oxidoreductase), 3rd (coenzyme Q:cytochrome *c* oxidoreductase), and 4th (cytochrome *c* oxidase) respiratory complexes and F_0_-ATP synthase. Characteristically, this decrease was partially compensated in the “0” rats (Supplementary Figure 2). It was previously shown that KM rats demonstrated reduced ATP production and increased H_2_O_2_ levels in the brain and liver compared with W rats (Venediktova et al., 2017). In our study, KM rat mitochondrial disorders also induced an increase in the expression of a number of genes that positively regulate apoptosis (Figure 4). An increased apoptosis rate in the inferior colliculi in KM rats compared to W was previously described (Chernigovskaya et al., 2018). This increase in apoptosis rate may be associated with abnormal brain development in KM rats, which in turn may be the cause of epileptogenesis, this effect was experimentally demonstrated in inferior colliculi and hippocampus of KM rats (Chernigovskaya et al., 2018; Kulikov et al., 2020). The delay in IC development was also demonstrated, in line with apoptosis, (Kulikov et al., 2020).

In addition to mitochondrial disorders, the reduced expression of the *Msh3* gene and an increase in DNA repair disorders may elevate the frequency of mutations and hence contribute to the rapid selection of the audiogenic phenotype. Similarly, it was previously shown that the 101/HY mouse strain, which is characterized by intense AE, is hypersensitive to chemical mutagens (these mice carry a mutation in the locus controlling DNA repair after chemical mutagenesis) (Boiarshinova et al., 2009). The mechanisms of reducing the expression of the *Msh3* gene in KM rats need further study.

An important link in the process of AE development is the increase in MAPK/ERK1/2 activity. We showed the overexpression of a number of MAPK cascade genes and increased phospho-ERK1/2 levels in KM rats (Figures 1, 3) in comparison to both W and “0” strain rats. A decrease in the expression level of some MAPK cascade genes in “0” rats can be considered as a compensatory effect, leading to a decrease in sensitivity to loud sound. The proapoptotic (and presumably proinflammatory) responses in the KM rats may have been connected to the increased transcription of a number of genes coding the upstream components of the JNK/p38 cascade (IL1R, TGFBR, and CD14) that are critical for these reactions (Verstrepen et al., 2008). In KM rats the apoptotic events were described in Chernigovskaya et al. (2018). The upregulation of inflammatory pathways in neuronal tissue was shown to have a crucial effect in the development of epilepsy (Rana and Musto, 2018). To our knowledge no current data on the KM strain inflammation system exist. In addition, KM rats have been shown to exhibit a significant increase in the expression of *GADD45* genes, which are involved in the activation of p38 and induction of apoptosis in response to DNA damage, which may be associated with impaired mitochondrial function, increased H_2_O_2_ production and inflammation (Liebermann and Hoffman, 2002). In our study, KM rats exhibited an increase in the transcription of growth factor genes and genes encoding the receptor tyrosine kinases PKC and RasGRP, which may have led to the increase in the activation of ERK1/2. On the other hand, the *Ras* transcription level was found to decreased in the KM rats, which may have compensated for the increase in upstream components in the system (Figure 4). Notably, the increase in the phosphorylation level of ERK1/2 in the KM rats (in comparison with the W and “0” rats) was directly shown in the present work. Previously, elevated levels of phospho-ERK1/2 were demonstrated in the inferior colliculi, hippocampus and striatum of KM rats (Dorofeeva et al., 2017; Chernigovskaya et al., 2018, 2019). We assume that activation of ERK1/2 may be a key mechanism for the development of AE. ERK1/2 activity contributes to a decrease in the seizure threshold via mechanisms involving enhanced glutamatergic synaptic transmission, which is induced *via* the expression of glutamate excretion (VGLUT2) and subsequent stimulation of NMDA receptors, as well as by an increase in NMDA receptor number (Nateri et al., 2007; Doyle et al., 2010; Gangarossa et al., 2011, 2019). On the other hand, it was previously demonstrated that activation of glutamate via NMDA and AMPA and kainate receptors during seizures leads to an increase in ERK1/2 activity, which causes suppression of the functional activity and sensitivity of GABAA receptors in the granular cells of the dentate gyrus in the hippocampus, making the entire hippocampus susceptible to seizures and status epilepticus development (Kapur, 2018). A correlation between changes in NMDA and ERK1/2 kinase levels was previously shown by exploring the pharmacological glutamate receptor NR2B subunit inhibitor ifenprodil, which blocks ERK-induced seizures (Nateri et al., 2007). It was also demonstrated that inhibition of ERK1/2 by intraperitoneal injections of SL327 (a selective MEK1/2 inhibitor) blocked AE seizures in KM rats (Glazova et al., 2015).

Another characteristic feature of the KM strain that is lacking in the W and “0” strains is increased expression of the *Ttr* gene (Figure 5), which was previously shown to encode a protein called transthyretin (Soprano et al., 1985). Mutations in this gene in humans cause family amyloid polyneuropathy characterized by amyloid formation, slowly progressive dementia, seizures, ataxia, and subarachnoid hemorrhage (Ando et al., 2013; Franco et al., 2016). Several studies reported the neuroprotective role of transthyretin in Alzheimer’s disease and in cases of cobalt-induced seizures in animals (Stein et al., 2004; Kajiwara et al., 2008; Li and Buxbaum, 2011). These findings suggested that overexpression of the *Ttr* gene in KM rats may be a compensatory mechanism. On the other hand, transthyretin regulates the activity of GABAA receptors, which play an important role in the control of predisposition to seizures (Zhou et al., 2019). Importantly, high levels of *Ttr* expression and transthyretin protein and mutations in the *Ttr* gene were demonstrated in GASH/Sal hamsters and in WAR rats. To this end deficiency of GABAA receptor-mediated neurotransmission were previously shown in GASH/Sal hamster and WAR rat models (Damasceno et al., 2020; Díaz-Casado et al., 2020).

Finally, we found significant differences in the expression of several other genes in KM rats (vs W rats) that were compensated in the “0” rats and, according to available data, may be associated with the development of AE. We have shown that in KM rats, compared with W and “0” rats, there was a decrease in the transcription of the *Igfbp5* and *Gstm1* genes, the mutations of which were shown to increase the risk of developing epilepsy in humans (Reddy, 2004; Jamali et al., 2006; Ercegovac et al., 2015; Prabha et al., 2016). The *Gstm1* gene encodes glutathione S-transferase, which is an important component of the antioxidant defense system in the brain. Notably, disturbances in the antioxidant system in the KM rat brain, along with effectiveness of the respective therapy, were previously demonstrated (Fedotova et al., 1990; Medvedev et al., 1992). In addition, in our study, changes in the expression of genes coding K-channel regulatory subunits Kcne2 and Kcne5, as well as Cacng4, which regulates the activity of L-type Ca-channels, were found in KM rats, and these changes were compensated in the “0” strain (Figure 5). In some cases, epilepsy is considered “channelopathy” because it involves K- and Ca-channel anomalies in animal models and human epilepsy (Biervert et al., 1998; Lenzen et al., 2005; Uehara et al., 2008; Poolos and Johnston, 2012; Snowball et al., 2019), although no detailed analysis of the roles of these subunits in human epilepsy has been reported. Finally, changes in dopamine synthesis and type I interferon signaling genes expression, detected in “0” strain relative to KM may be crucial for compensation of AE proneness (Rana and Musto, 2018; Akyuz et al., 2021). However, there were no significant changes in dopamine receptors in KM and “0” strains relative to Wistar strain.

In summary, in rodents with AE phenotypes with different genetic origins, similar patterns of the expression of certain genes were found. In particular, an increase in *Ttr* transcription and mutations of the *Msh3* gene were previously described (Damasceno et al., 2020; Díaz-Casado et al., 2020). These gene expression peculiarities were associated with distinct pathological traits in brain function. On the other hand, other studies showed no changes in the brains of KM rats, concerning the expression of other genes characteristic of WAR and/or GASH/Sal models (*Npy*, *Egr3*, and *Rgs2*) while authors (Damasceno et al., 2020) associated these genes with a predisposition to AE in these animals. Additionally, in KM rats (as in rats of other AE-prone strains), no changes in the expression level of the *Vlgr1* gene were reported, while mutation of this gene caused the development of AE in Frings mice (Klein et al., 2005). In general, the data obtained confirm the polygenic inheritance of AE, as previously demonstrated by a diallel crossing experiment (Poletaeva et al., 2017). A few peculiarities of the gene expression profile in AE-prone animals relative to controls – AE-resistant strains – are common to two rodent species (rat and hamster), while other features are inherently “individual” to certain epileptic rat strain models. The expression of certain genes in the brains of animals with audiogenic epilepsy differs even among different strains of AE-prone rats, which may be associated with peculiarities of the genetic “background” of the original populations together with events during the selection process, among other causes. Notably, a number of gene expression characteristic traits in the brains of animals with AE, found both in this study and previously in the WAR strain, were confirmed in the GASH/Sal hamster strain (Damasceno et al., 2020; Díaz-Casado et al., 2020). This finding probably indicates that these genes represent certain “nodes” in brain pathology common to different AE genotypes.

## Data Availability Statement

The datasets presented in this study can be found in online repositories. The names of the repository/repositories and accession number(s) can be found below: https://www.ncbi.nlm.nih.gov/search/all/?term=GSE173885.

## Ethics Statement

The animal study was reviewed and approved by the Commission on Biomedical Ethics of the FSBI V. V. Zakusov Research Institute of Pharmacology (protocol No. 1 of January 31, 2020). The organization and execution of experimental work were carried out in accordance with the order of the European Convention for the Protection of Vertebrate Animals (Strasbourg 1986) and the Order of the Ministry of Health of the Russian Federation April 1, 2016, No 199N.

## Author Contributions

LC, SF, and AR: RNA-seq, Q-RT-PCR, and bioinformatic analysis. AD and DG: western blot analysis. IF and IP: working with animals. SF, ME, SL, and DG: experiment design and writing a manuscript. All authors contributed to the article and approved the submitted version.

## Conflict of Interest

The authors declare that the research was conducted in the absence of any commercial or financial relationships that could be construed as a potential conflict of interest.

## Publisher’s Note

All claims expressed in this article are solely those of the authors and do not necessarily represent those of their affiliated organizations, or those of the publisher, the editors and the reviewers. Any product that may be evaluated in this article, or claim that may be made by its manufacturer, is not guaranteed or endorsed by the publisher.

## Footnotes

1
https://ggplot2.tidyverse.org

## Supplementary Material

Supplementary Figure 1 Distribution of compensation coefficient for differentially expressed genes in the KM and W rats. The histogram shows the compensation coefficients for expression changes in the “0” rats. Density distribution was estimated using kernel density estimation (KDE). The compensation coefficient was calculated as a percentage of mean (logCPM) KM – mean (logCPM) 0)/(mean (logCPM) KM – mean (logCPM) W.

Supplementary Figure 2 The expression of genes involved in the oxidative phosphorylation pathway was partly attenuated in the “0” rats. **(A)** Differential expression of genes participating in the oxidative phosphorylation pathway (KEGG rno00190). The illustration of the pathway is shown with slight modifications. Gene expression changes are shown in color (log2-scale; LogFC; red – upregulation, blue – downregulation, gray – no change, white background – the absence of expression). Each gene block is divided into two sections (the left block shows differentially expressed genes in the KM and W rat samples; the right block shows the differentially expressed genes in the “0” and KM rat samples). **(B)** Scatter plot depicting the pairwise comparison of log2-fold changes between the KM vs W rat and “0” vs KM rat groups. R – Spearman’s correlation coefficient.

Supplementary Table 1 Primers for Q-RT-PCR used in the work.

Supplementary Table 2 Differentially expressed genes (DEG) between all groups of comparison.
