# Supplementary figures and images for: Transcriptome of the Krushinsky-Molodkina Audiogenic Rat Strain and Identification of Possible Audiogenic Epilepsy-Associated Genes
Source: Front Mol Neurosci. 2021 Nov 4;14:738930. doi: 10.3389/fnmol.2021.738930 (PMC8600260; doi:10.3389/fnmol.2021.738930)

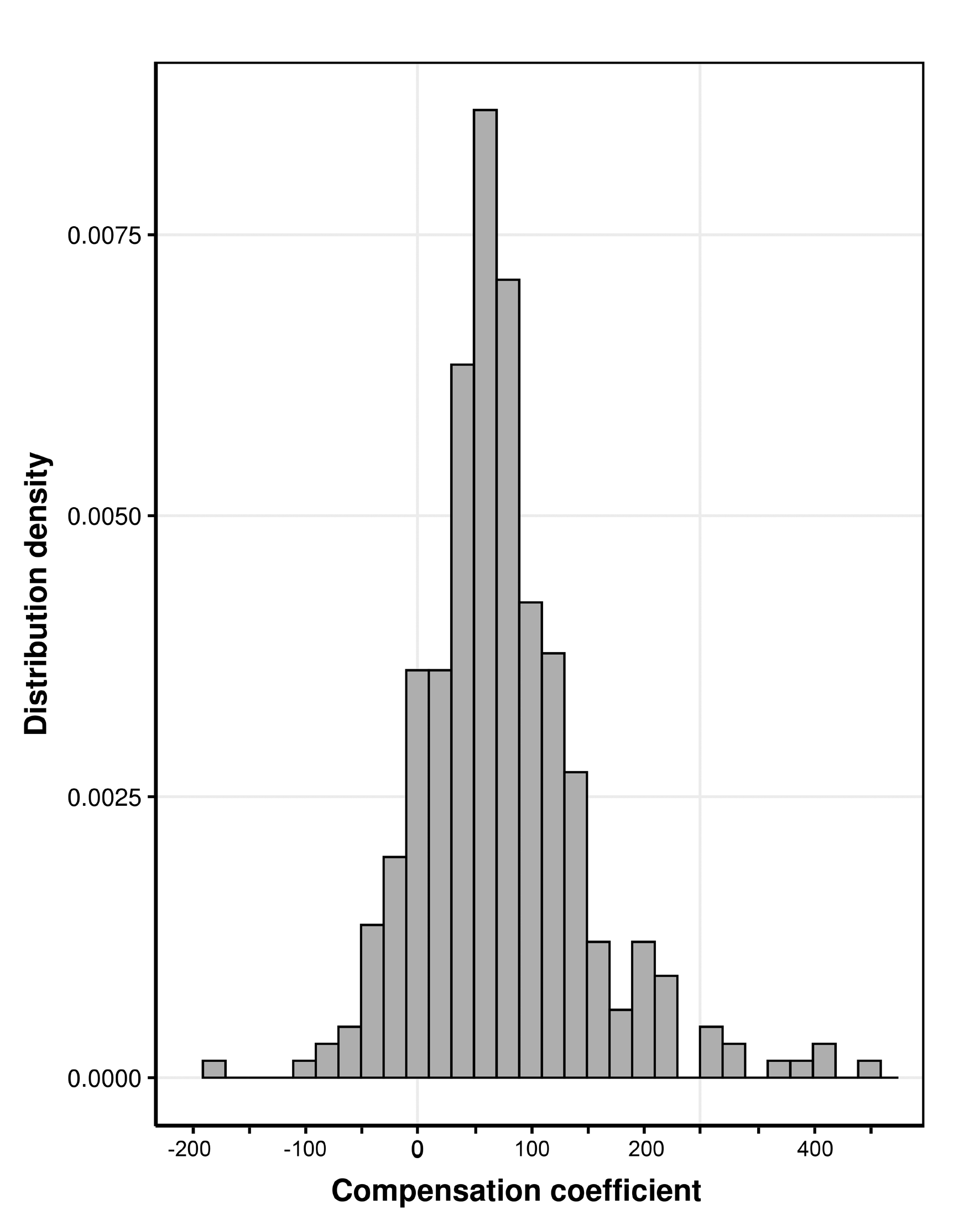

Supplement: Supplementary Figure 1 — Distribution of compensation coefficient for differentially expressed genes in the KM and W rats. The histogram shows the compensation coefficients for expression changes in the “0” rats. Density distribution was estimated using kernel density estimation (KDE). The compensation coefficient was calculated as a percentage of mean (logCPM) KM – mean (logCPM) 0)/(mean (logCPM) KM – mean (logCPM) W. [file Image_1.TIF]

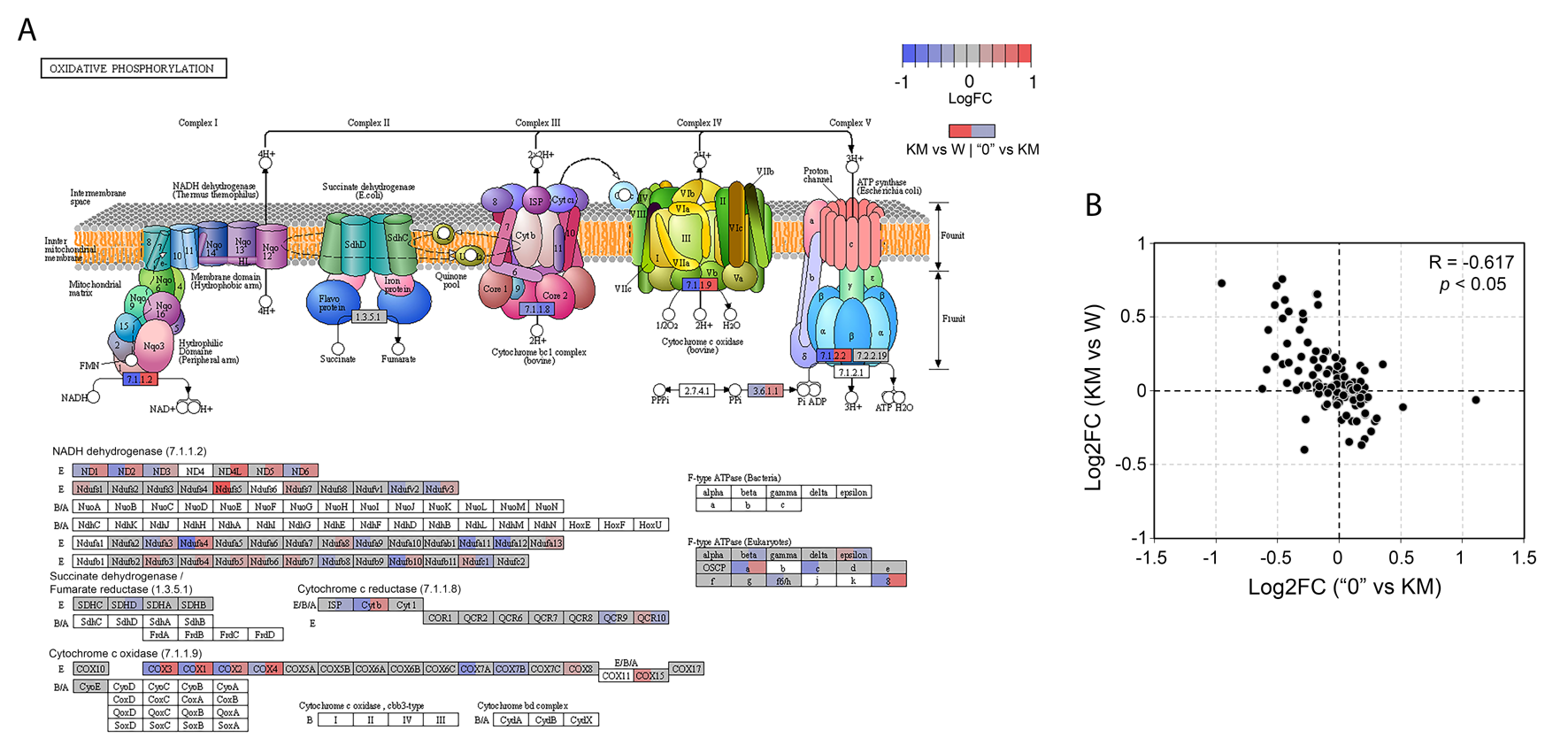

Supplement: Supplementary Figure 2 — The expression of genes involved in the oxidative phosphorylation pathway was partly attenuated in the “0” rats. (A) Differential expression of genes participating in the oxidative phosphorylation pathway (KEGG rno00190). The illustration of the pathway is shown with slight modifications. Gene expression changes are shown in color (log2-scale; LogFC; red – upregulation, blue – downregulation, gray – no change, white background – the absence of expression). Each gene block is divided into two sections (the left block shows differentially expressed genes in the KM and W rat samples; the right block shows the differentially expressed genes in the “0” and KM rat samples). (B) Scatter plot depicting the pairwise comparison of log2-fold changes between the KM vs W rat and “0” vs KM rat groups. R – Spearman’s correlation coefficient. [file Image_2.TIF]
