# Supplementary material for: Transcriptome of the Krushinsky-Molodkina Audiogenic Rat Strain and Identification of Possible Audiogenic Epilepsy-Associated Genes
Source: Front Mol Neurosci. 2021 Nov 4;14:738930. doi: 10.3389/fnmol.2021.738930 (PMC8600260; doi:10.3389/fnmol.2021.738930)
Supplement: Supplementary Table 1 — Primers for Q-RT-PCR used in the work. [file Table_1.docx]

Supplementary table S1. Primers for Q-RT-PCR used in the work.

| Gene | Sequence |
| --- | --- |
| *Msh3* | GCTAGCAGATGTGCCCAGAGA  ACGTCTGTATTTCCTGTATTTCC |
| *Ttr* | ACGGAAGGGGTGTACAGGGT  TGCGATGGTGTAGTGGCGATG |
| *Acsm5* | CAGCCCAGACCCCATCCG  ACCGTCTTTGGCAGTTCCGA |
| *Kcne2* | ATCTTCGTCCAATCGTGCGTG  CCTGTTGTTCGGCTGTTGTG |
| *Kcne5* | TCTTGAACCGCTTGCTGCTGG  CTTGCGGGAGCGAGTGTAGG |
| *Cacng4* | ATTGTGCGAGCCTCCAGTGTC  TGATGCCGATGATATTACTGAGG |
| *Ywhaz* | TTGAGCAGAAGACGGAAGGT  GAAGCATTGGGGATCAAGAA |
